# Aerobic Exercise and Healthy Nutrition as Neuroprotective Agents for Brain Health in Patients with Parkinson’s Disease: A Critical Review of the Literature

**DOI:** 10.3390/antiox9050380

**Published:** 2020-05-05

**Authors:** Davide Maria Cammisuli, Ubaldo Bonuccelli, Simona Daniele, Claudia Martini, Jonathan Fusi, Ferdinando Franzoni

**Affiliations:** 1 Neurology Unit, Santa Chiara Hospital, Department of Clinical and Experimental Medicine, University of Pisa, 56126 Pisa, Italy; ubaldo.bonuccelli@unipi.it; 2 North-Western Tuscany Region, National Health System, Local Health Unit, 56126 Pisa, Italy; 3 Department of Pharmacy, University of Pisa, 56126 Pisa, Italy; simona.daniele@unipi.it (S.D.); jonathan.fusi@gmail.com (J.F.); 4 Department of Clinical and Experimental Medicine, University of Pisa, 56126 Pisa, Italy; claudia.martini@unipi.it

**Keywords:** Parkinson’s disease, aerobic exercise, diet, nutrition, rehabilitation

## Abstract

Parkinson’s disease (PD) is characterized by motor and nonmotor features that have an influence on patients’ quality of life at different levels. To date, some evidences have arisen on the effectiveness of physical trainings and nutrients intake in ameliorating functional and cognitive outcomes in PD patients. Physical activity is effective in improving both motor and nonmotor features and recent epidemiological investigations have revealed the pivotal role that dietary patterns may play in reducing the risk of PD highlighting the pathogenesis of the neurodegeneration. Specifically, aerobic exercise shows beneficial effects in improving motor functions and executive control in PD patients, as well as proper nutrition may help in improving neuroprotective agents counteracting neurodegeneration and allows patients to better interact with the medication. Our narrative review critically focused on aerobic exercise and nutrition in PD in order to point out the best prescriptions for brain health of affected patients. Implications for a therapeutic plan and rehabilitation for these patients are also discussed.

## 1. Parkinson’s Disease (PD): Epidemiology and Clinical Features 

PD represents the second most prevalent neurodegenerative disorder that affects 2–3% of the population over 65 years of age [1], and 4–5% of those people above the age of 85 [2]. It is characterized by the degeneration of dopaminergic neurons of the *substantia nigra* (which causes a striatal dopamine deficiency and functional impairment of some brain circuits, including motor ones) and intracellular inclusions containing aggregates of α-synuclein [3]. 

Depletion of dopaminergic neurons projecting from the *substantia nigra* to the dorsal striatum results in the aetiology of the cardinal motor symptoms of PD (i.e., bradykinesia, resting tremor, rigidity) [4]. Molecular pathogenesis of PD includes mitochondrial function and oxidative stress, calcium homeostasis, axonal transport, and neuroinflammation [1]. More specifically, mitochondrial activity disturbances in energy metabolism increase the production of reactive oxygen species (ROS) leading to oxidative stress and neuronal degeneration [5]. The critical role played by the gut microbiota—consisting of thousands of bacterial species—has been newly debated since it is linked to intestinal barrier integrity, metabolism, immunity, and brain functioning [6] in several neurodegenerative conditions, such as PD. An outstanding recent investigation, exploring the contribution of the gut microbiota on the behavioral and neurochemical alterations in a rodent toxin model of dopamine depletion reproducing PD-associated motor symptoms, concluded that the gut microbiota represents a potential contributor for PD pathology [7]. 

PD diagnosis relies on the cardinal motor features, but the disease may be associated with different nonmotor symptoms (i.e., cognitive impairment, neuropsychiatric symptoms, sleep disorders, and sensorial dysfunction) that compromise patients’ clinical status, negatively impacting on quality of life (QoL) and are significantly associated with reduced wellbeing [8]. Specifically, despite PD being originally classified as a movement disorder, cognitive problems are present in a large percentage of PD patients, approximately 30% to 40% [9]. They mainly include deficits in attention, visuospatial, and constructive abilities [10]. In addition to these deficits, a wide range of executive functions (EF) pertaining overall executive abilities, working memory, planning, inhibitory control, and set-shifting are impaired in PD [11,12]. EF refer to higher cognitive processes that regulate goal-directed behavior [13] and are based in the dynamic interaction between the prefrontal cortex and other cortical and subcortical regions [14]. EF deficits are common in PD and have been attributed to basal ganglia-thalamus-cortical circuitries disruption. Moreover, EF outcomes are variable in their dopamine-response treatment for nigrostriatal-related symptomatology [15]. 

Although of idiopathic origin, genetic causes and environmental factors are also recognized as important triggers of the disease. Less than 10% of PD is associated with specific genetic changes, and diet represents one of the environmental factors that may promote or exacerbate PD progression [16,17]. Dietary factors are difficult to interpret in the estimation of PD risk. To this end, some researches have pointed out how reduction of calories intake during life is associated with a more extended life span and an improvement of brain functioning and overweight in middle life has been identified as a key risk factor for PD [18] Accordingly, the risk of developing such a neurological condition appears to be inversely associated with physical activity practiced during life [19,20,21]. Such a risk seems to be mediated by other factors than cardiovascular and/or metabolic ones [22]. However, it results in being more attenuated in people who regularly perform moderate to vigorous activities, but not in those performing light activities [23].

Beyond pharmacological (i.e., levodopa, carbidopa, dopamine agonists, MAO-B inhibitors, catechol O-methyltransferase, anticholinergics, and amantadine) and nonpharmacological treatments (e.g., cognitive trainings, neurostimulation, occupational therapy) [4], there is an urgent need to encourage healthy lifestyles in people with PD, such as dietary habits and physical activity for counteracting motor dysfunction and ameliorating brain health. Recently, some researchers have carefully explained the role of physical exercise in PD [24,25,26], whereas that played by nutrition appears less investigated in the literature. Starting from this assumption and in line with the fact that a multidimensional treatment constitutes the best way to counteract the aging process and related neurological conditions, the authors of the present study have particularly focused both on aerobic exercise and healthy nutrition as the most relevant factors influencing brain health. 

## 2. PD and Aerobic Exercise

Aerobic exercise (AE) as the activity using large muscle groups, rhythmic in nature (e.g., cycling, dancing, hiking, jogging, running, swimming, and walking) per se [27] may help in preventing cardiovascular disease, reducing high-density lipoprotein cholesterol (i.e., HDL-C) and adipose tissue distribution, increasing insulin sensitivity, improving executive functions, enhancing response to psychosocial stressors, and even exerting a detrimental effect on depression [28]. 

AE promotes brain health by reducing inflammation and oxidative stress and stabilizing calcium homeostasis [29]. Some studies have estimated that a significant 60% lower PD risk in men and a lesser percentage in women is associated to moderate-to-vigorous physical exercise in early adulthood [30,31]. Physical activity in PD is related to increased insulin/insulin-like growth factor (IGF) sensitivity and ketone utilization, improved expression of brain-derived neurotrophic factor (BDNF), fibroblast growth factor (FGF2), and vascular endothelial growth factor (VEGF) with enhanced bioenergetics and neuroplasticity [32]. 

Investigations in rat models have shown that aerobic exercise may trigger plasticity-related changes in the central nervous system (CNS) including synaptogenesis, enhanced glucose utilization, angiogenesis, and neurogenesis [33]. In particular, Wang et al. [34] firstly demonstrated in a rat model of intra-striatal 6-hydroxydopamine (6-OHDA)-induced parkinsonism that four weeks of a forced running wheel exercise improves motor deficits and results in altered regional brain activation including motor cortex, caudate, putamen, globus pallidus, zona incerta, and cerebellum [34], and secondarily [35] described the effectiveness of a long-term aerobic exercise on functional connectivity of motor circuits, by finding that long-term exercise training partially reversed lesion-induced alterations in rsCF of the motor circuits and enhanced functional connectivity in specific motor pathways in the parkinsonian rats, which could underline recovery in motor functions observed in these animals. Moreover, evidence of angiogenesis following a four-week program of treadmill training has been reported in the brain of chronic parkinsonian mice [36]. 

To date, some authors have investigated the effects of AE on cognitive/motor skills in PD patients. For instance, Nadeau et al. [37] demonstrated that an intervention of 72 one-hour sessions over 24 weeks of treadmill training in PD patients with a mild motor dysfunction was conducted to an improvement in global cognition and QoL. Tanaka et al. evaluated the AE effect on EF in PD [38]. In this study, a small group of participants (*n* = 20) was equally assigned to an experimental group following a generalized AE training for six months, while a control group followed the usual care. Such an intervention was able to enhance EF after controlling for confounding variables (i.e., attention, anxiety, and depression). Cruise et al. [39] compared an experimental group (*n* = 15) of PD patients that underwent strength and cardiovascular training involving twice weekly exercise sessions of approximately 60-min duration and a control group (*n* = 13) with the same condition maintained its usual lifestyle. AE has been shown to enhance specific EF abilities (i.e., spatial working memory and verbal fluency, both semantic and category) compared to cognitive tasks mainly mediated by the temporal lobe (i.e., spatial recognition memory and pattern recognition memory). Reuter et al. [40] have demonstrated that walking and Nordic walking training can improve stride length, gait variability, maximum walking speed, exercise capacity at submaximal levels, and ameliorate motor scores on the Unified Parkinson’s Disease Rating Scale (i.e., UPDRS) in a group of PD patients with moderate motor symptoms. Nordic walking has been also proven as a sustaining brain function associated to movement preparation [41], motor and nonmotor functions, fatigue, apathy and depression [42], functional mobility, and associated parameters [5]. Further, Duchesne et al. [43] have assessed the effect of an aerobic exercise intervention (i.e., bike-training program), three times a week for 12 weeks on EF and implicit motor sequences learning in a group of 19 early PD individuals compared with 20 healthy controls. Such a kind of training improves inhibition and motor learning skills. Moreover, Picelli et al. [44] conducted a randomized single-blind pilot trial on 17 patients with mild to moderate PD of which nine patients were allocated to the intervention group receiving 45-min sessions of treadmill training (one session a day, three days a week), while eight patients were allocated to the control group with regular social interaction. One month later, patients allocated into the experimental group significantly improved in global executive functions and walking capacity. Finally, Koop et al. [45] have demonstrated that mobility—often associated with falls in PD patients—improves after an eight-week training of high intensity AE. 

Recently, da Silva et al. [25] documented that combined cognitive and motor training (i.e., treadmill training performed three times a week for a period of two months) promote significant effects on global cognition and on specific abilities of the attention system and executive functioning in patients with a six-year diagnosis of PD. Accordingly, walking on the treadmill may be used as an easy and accessible way to improve stride length and balance in PD patients [46], and an intensive treatment (i.e., 72 one-hour exercise sessions for 24 weeks) can increase speed and endurance in PD patients with a six-month maintenance effect [44]. 

**Figure 1:**
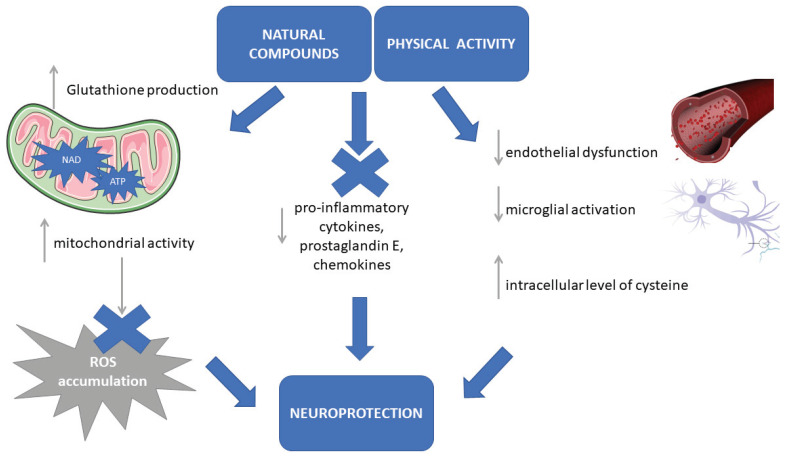
Neuroprotective effects of aerobic exercise and natural antioxidant compounds. The neuroprotective effects of physical activity and antioxidant compounds are related to (i) the production of glutathione that increases mitochondrial activity and prevents reactive oxygen species (ROS) accumulation; (ii) the increase of mitochondrial activity through nicotinamide adenine dinucleotide (NAD) biosynthesis and ATP production; (iii) the reduction of the pro-inflammatory molecules; (iv) the reduction of endothelial dysfunction and the inhibition of microglial activation, the increasing of intracellular level of cysteine.

The neurotrophins that are associated with regular physical activity are able to stabilize intracellular calcium concentration, induce antioxidant enzyme expression, and suppress the release of pro-inflammatory cytokines [47]. Moreover, physical exercise can decrease endothelial dysfunction (Figure 1). Specifically, physical activity may reduce the alteration of the dopaminergic neurons in the *substantia nigra* and helps in reconstructing basal ganglia functions involved in motor commands [48]. Aerobic exercise (performed five days a week for four weeks) in parkinsonian rats on sessions lasting from 20 to 60 min can restore the expression of glial fibrillary acidic protein in the dorsal striatum [49] and regular and continuous aerobic training of rats over a period of 18 months was reported to show a neuroprotective effect on the cerebellum, a brain structure that is centrally involved in movement and balance control [50]. 

## 3. PD and Nutrition

In the last years, an increasing attention has been paid on the patient’s diet, use of food supplements, and food rich in vitamins and antioxidants, as complementary elements relevant for prevention and treatment of some neurological conditions such as PD [51].

In the Rotterdam Study, a cohort of 5.289 individuals of 55 years of age and older without dementia was prospectively studied over six years by medical monitoring. Researchers discovered that a high intake of unsaturated fatty acids and vitamin B_6_ might protect against PD [52,53]. Conversely, Logroscino et al. [54], by an in-depth analysis of dietary pattern, medical history, and lifestyle practices on 47.406 men and 76.947 women from the USA, did not find an association between total iron intake (dietary and supplemental) and risk of PD. Another relevant study by Ross et al. [55], conducted by analyzing data from a 30-year follow-up of 8.004 Japanese-American men aged (45–68 years), has pointed out that higher coffee and caffeine intake was associated with a lower incidence of PD as a mechanism independent of smoking. Further, Park et al. [56] documented the relationship between milk intake and subsequent risk of PD, in a cohort of 7.504 men (aged 45–68), which biometrical data were pooled for the *Honolulu Hearth Programme* following them for 30 years, as well. A large prospective study including 49.692 men and 81.676 women, i.e., the Health Professionals Follow-up Study and the Nurses’ Health Study (1984–2000) conducted by Gao et al. [57,58], concluded that the dietary pattern with a high intake of berry fruits, apples, oranges, vegetables, legumes, whole grain, nuts, fish and poultry, flavonoid-rich food (e.g., tea, red wine), and a low intake of saturated fat and alcohol may protect against PD. 

Finally, Ciulla et al. [59] showed that some molecules and natural compounds may have different beneficial effects in PD patients, such as (i) antioxidant ones (i.e., coenzyme Q10, lipoid acid, *N-acetyl-cysteine*, vitamin E, carvacrol, curcumin, omega-3, whey protein, vitamin D_3_, creatine, melatonin, niacin, vitamin C, 6-shogaol, *beta*-carotene, lycopene), (ii) anti-inflammatory ones (i.e., lipoid acid, carvacrol, curcumin, fatty acids, 6-shogaol, quercetin), (iii) of neuroprotection (i.e., coenzyme Q10, lipoid acid, *N-acetyl-cysteine*, curcumin, vitamin D_3_, creatine, vitamin B_3_, 6-shogaol, epigallocatechin-3-gallate), and (iv) of neuromodulation (i.e., ginkgo biloba extract, carvacrol). 

**Figure 2:**
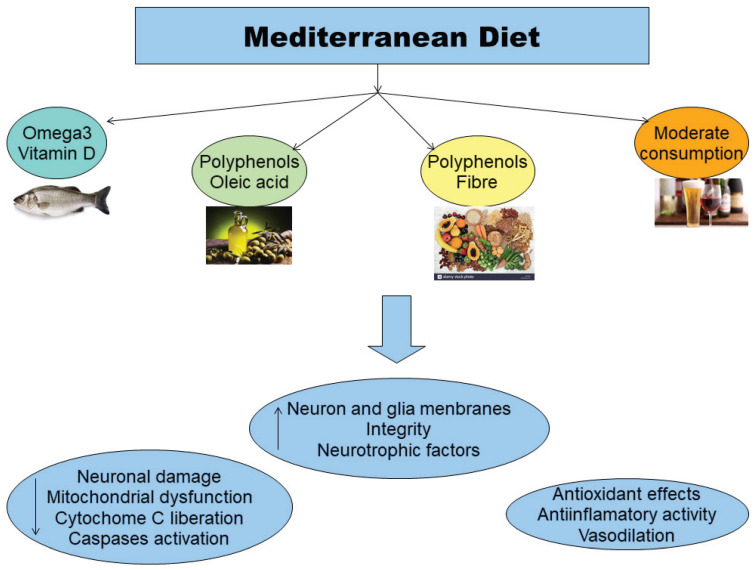
Beneficial effects of the Mediterranean diet. The main constituents of the Mediterranean diet (i.e., “good fats”, vitamins, polyphenols, phytosterols, and carotenoids), provide an equilibrated mix of nutrients with antioxidant, anti-inflammatory, and prebiotic effects.

In this context, good nutrition provided by the Mediterranean diet has been established as a powerful tool to modulate the systemic inflammatory balance by slowing down the age- and neurodegeneration-related increase in the production of inflammatory molecules and by favoring an adaptive anti-inflammatory response [60]. Indeed, the Mediterranean diet is characterized by the consistent intake of “good fats”, vitamins, polyphenols, phytosterols, and carotenoids, providing an equilibrated mix of nutrients with antioxidant, anti-inflammatory, and prebiotic effects (Figure 2). A novel randomized controlled trial [61] has shown that PD patients undergoing the Mediterranean diet regimen remarkably increase the cognitive domains of executive functions, language, attention and concentration, and memory. Adherence to the Mediterranean diet has been associated with a significant lower probability of prodromal PD in older people [62]. Further studies are needed to elucidate the underlying neurobiological mechanisms.

## 4. Neuroprotective Effects of Aerobic Exercise and Balanced Diet in PD

The implementation of physical activity programs for people with PD has resulted in beneficial effects for autonomy in daily life activities, motor tests scores, and functional independence [63]. According to the previous relevant studies [64], the greatest neuroprotective effects of aerobic exercise appear to be related to prefrontal areas of the brain, which support executive functions and attentional control [65] that are frequently impaired in PD [12]. Moreover, AE enables PD patients to maintain their psychomotor learning abilities [66], too. A significant study using fMRI examined the effect of AE training (i.e., a bike program performed three times per week for 12 weeks), showing for the first time an increasing functional activity in the striatum of PD patients and its strict correlation to aerobic fitness [67]. 

Molecular mechanisms behind the beneficial effects of motor interventions in humans are limited per se, and the most relevant conclusions are derived from animal studies pointing out the central role of neurotrophic factors in the protection of dopaminergic neurons, by increasing the sprouting of the residual dopaminergic axons in the striatum or the generation of local striatal neurons from inhibitory interneurons [32]. 

In the last decades, diet has emerged as a critical lifestyle factor that may prevent or slow down PD symptoms progression [68]. In particular, adherence to the Mediterranean diet is associated with a lower probability of developing PD [69,70], given that it is rich in fresh vegetables and fruits, nuts, seeds, fish, olive oil, fresh herbs, and spices conversely to the Western diet that is considered among the greatest risk factor for developing PD [71], because it is rich in caloric foods, saturated and omega-6 fatty acids, excessive salt and refined sugar, low intake of omega-3 fatty acids, and fiber. 

Moreover, some molecules and natural compounds able to mitigate pathophysiological mechanisms characterizing PD, should be thought as balancing complements of dietary regimens. Specifically, their effects are related to (i) the production of glutathione that reduces oxidative stress, increases mitochondrial activity, and prevents ROS accumulation (i.e., antioxidant), (ii) the reduction of the pro-inflammatory cytokines, prostaglandin E, and chemokines mediating inflammatory cascade (i.e., anti-inflammatory), (iii) the modulation of mitochondrial activity in neurons and biosynthesis of nicotinamide adenine dinucleotide (NAD) for ATP production (i.e., neuromodulation), (iv) the influence on cell survival and mitochondrial activity, the reduction of endothelial dysfunction and the inhibition of microglial activation, the increasing of intracellular level of cysteine (i.e., neuroprotection), respectively [68]. The molecular mechanisms relative to some molecules and natural compounds in mitigating the pathophysiological mechanisms characterizing PD are reported in Figure 1.

## 5. Implications for PD Therapeutic Plan and Rehabilitation

In addition to motor disability, a set of sensorimotor (i.e., anosmia, pain, paraesthesia), autonomic (i.e., dysphagia, constipation, urinary incontinence), behavioral (i.e., depression, apathy, anxiety, emotional liability, and impulsivity) [72], and cognitive deficits (particularly, executive ones) [73] may occur in the course of the PD and severely influenced QoL [74]. On 13 June, 2019, *the American Parkinson’s Disease Association* has declared results of the study, entitled “*The Economic Burden of Parkinson’s disease*” in the USA, by stating that $52 billion per year is spent on PD-related expenses. Despite the fact that naturally drugs therapy represents the gold standard for the patient’s motor symptomatology, it may cause several side effects, such as hypotension, nausea, dyskinesia, and psychiatric symptoms [75]. Considering these side effects as well as sanitary costs, complementary strategies such as AE and nutrition are necessary with the final aim of improving patients’ wellbeing. 

According to Nocera et al. [76], AE is relatively inexpensive, noninvasive, and unlikely to interact with other treatments, and can effectively improve the physical and mental wellbeing of PD patients. Of course, interventions should be tailored to the specific needs of patients and care should mainly take into consideration potential falls risk and comorbid symptomatology reported by some of them. 

Currently, there is also consistent evidence that some nutrients such as omega-3, vegetables, fruits, carotenoids, genistein, tea, caffeine, and resveratrol promote neuroprotection while others (i.e., milk) may increase the risk for PD [77] or interfere with the medication. The role of the intestinal microbiome in PD has been recently claimed as a viable approach to prevent or modify disease progression. Jackson et al. [69] have reviewed studies reporting evidence for the dysregulation intestinal microbiome in PD characterized by a loss of short fatty acids (SCFA) bacteria and increased lipopolysaccharide (LPS) bacteria. A decreased production of SCFA and an increased LPS have been reported to contribute to PD development/exacerbation. A nutrition from the Mediterranean diet decreases the risk for PD, thanks to the major abundance of SCFA-producing or LPF-containing bacteria in the intestinal microbiome with an effect on the organ barrier function, endotoxemia, NLRP_3_ inflammasone activation, insulin resistance, and mitochondrial dysfunction, and the production of glucago-like peptide 1 (GLP-1), BDNF, and intestinal gluconeogenesis [78].

Despite promising effectiveness of some physical trainings and nutrients in PD, large prospective randomized controlled trials combining interventions of AE and a balanced diet are urgently needed in the future, also in addition to cognitive trainings and neurostimulation [74]. 

According to Xu et al. [20], we cannot exclude that less participation in AE may constitute an early marker of PD in individuals at higher risk: Physical activities such as walking in community settings are safe, well-tolerated, and improve cardiovascular fitness and motor functioning [31], mood and executive control, as well as QoL of PD patients [79], and they can be remotely supervised as a home-based treatment [80], too. While disease severity, difficulties of gait, and disability in daily living predict part of the physical inactivity [81], a treadmill training performed three times a week for about 60 min should be practiced by PD patients for improving cognition, with positive implications for motor functions and mood [25].

Information of dietary pattern and practiced AE remain quite undetected in PD patients by clinicians in clinical practice and should be routinely integrated into the therapeutic plan. Constipation, dysphagia, depression, and hyposmia affecting patients with PD may influence dietary choices [82], as well as depression [83], apathy [84], and musculoskeletal pain [84] may have an effect on patients’ engagement in physical activity, especially aerobic ones. An evaluation including dietary pattern and lifestyles (i.e., aerobic exercise levels) beyond neurological examination, should be routinely adopted by clinicians as a multidimensional practice. To date, only a low percentage of PD patients routinely performed physical activity, especially aerobic ones. As recently reported by the Parkinson Progression Markers Initiative (PPMI) only 47% (of 383 PD patients enrolled in the study) confirm activity consistent with the American Heart Association (AHA) recommendation (i.e., 150 min of moderate exercise or 75 min of vigorous exercise weekly performed) [85]. Likewise, the majority of PD patients do not follow a specific diet prescription, so that therapeutic effects of medication and risk of PD-related symptomatology exacerbation can be successfully reduced. 

A medical history contemplating patients’ screening for coronary heart disease, hypertension, pulmonary functions, cardiovascular disease, decompensated diabetes, and osteoarthritis represents an essential element of the therapeutic plan. A comprehensive rehabilitation program tailored on patients’ definite needs with specific prescriptions on diet and AE scheduling should be adopted by health professionals’ teams including nutritionists, sport medicine clinicians, and even clinical psychologists for the implementation of care planning formulated by neurologists for PD patients. A summary of the main benefits for PD patients from AE and healthy nutrition is reported in Table 1. Beyond them, we would like to stress that PD patients may take advantage of integrated programs, also including cognitive trainings performed through paper-and-pencil or computerized tasks given that the best prescription for treating PD patients is constituted by these three pillars, as recommended by the Parkinson’s Foundation 2018 Guidelines [86].

## 6. Conclusions

AE and balanced diet currently represent complementary strategies to drugs treatment for counteracting motor and non-motor symptoms in patients with PD. Their administration along side to cognitive stimulation trainings may be routinely used in PD rehabilitation. Large randomized controlled trials with long-term follow-up to monitor maintenance effects using combined techniques are warrant to improve primary evidence in the next future.

## Figures and Tables

**Table 1:** Summary of specific and nonspecific effects of aerobic exercise and balanced diet on Parkinson’s disease (PD).

Neuroprotective Agents	Specific Effects	Nonspecific Effects
AE	Psychomotor learning abilities Protection of dopaminergic neurons	Ameliorating executive functions/attentional control
Balanced diet	Antioxidant Anti-inflammatory Neuromodulation Neuroprotection	Global enhancement of cognitive domains efficiency

Note: PD: Parkinson’s disease; AE: Aerobic exercise.
